# In the national epidemiological bulletins – a selection from current issues

**DOI:** 10.2807/1560-7917.ES.2016.21.35.30330

**Published:** 2016-09-01

**Authors:** Eurosurveillance editorial team

**Affiliations:** 1 European Centre for Disease Prevention and Control (ECDC), Stockholm, Sweden

**Keywords:** public health, Europe

## Sexually transmitted diseases

### Chlamydia 2015

EPI-NEWS 34, 2016

24 August 2016, Denmark

http://www.ssi.dk/English/News/EPI-NEWS/2016/No%2034%20-%202016.aspx

## Vaccine-preventable diseases

### Measles associated with summer festivals

Health Protection Report; 10(25)

5 August 2016, United Kingdom

https://www.gov.uk/government/publications/health-protection-report-volume-10-2016/hpr-volume-10-issue-25-news-5-august#measles-associated-with-summer-festivals

### National measles outbreak – April to July 2016

Epi-Insight 2016;17(8)

August 2016, Ireland

http://ndsc.newsweaver.ie/epiinsight/s7l9818i9vu?a=2&p=50630336&t=17517804

## Zoonoses and vector-borne diseases

### Leptospirosis: 2011-2016

EPI-NEWS 28-33, 2016

17 August 2016, Denmark

http://www.ssi.dk/English/News/EPI-NEWS/2016/No%2028-33%20-%202016.aspx

## Other

### Unusual case of neonatal herpes simplex virus infection

Epidemiologisches Bulletin 32, 2016

15 August 2016, Germany

https://www.rki.de/DE/Content/Infekt/EpidBull/Archiv/2016/Ausgaben/32_16.pdf?__blob=publicationFile

